# Strategies Used by Pet Dogs for Solving Olfaction-Based Problems at Various Distances

**DOI:** 10.1371/journal.pone.0131610

**Published:** 2015-07-15

**Authors:** Zita Polgár, Ádám Miklósi, Márta Gácsi

**Affiliations:** 1 Department of Ethology, Eötvös Loránd University, Budapest, Hungary; 2 MTA-ELTE Comparative Ethology Research Group, Budapest, Hungary; Université Lyon, FRANCE

## Abstract

The olfactory acuity of domestic dogs has been well established through numerous studies on trained canines, however whether untrained dogs spontaneously utilize this ability for problem solving is less clear. In the present paper we report two studies that examine what strategies family dogs use in two types of olfaction-based problems as well as their success at various distances. In Study 1, thirty dogs were tasked with distinguishing a target, either their covered owner (Exp 1) or baited food (Exp 2), from three visually identical choices at distances of 0m (touching distance), 1m, and 3m. There were nine consecutive trials for each target. We found that in Exp 1 the dogs successfully chose their owners over strangers at 0m and 1m, but not at 3m, where they used a win-stay strategy instead. In Exp 2 the dogs were only successful in choosing the baited pot at 0m. They used the win-stay strategy at 1m, but chose randomly at 3m. In Study 2, a different group of dogs was tested with their owners (Exp 1) and baited food (Exp 2) at just the 3m distance with two possible targets in 10-10 trials. In Exp 1 the dogs’ overall performance was at chance level; however, when analyzed by trial, we noticed that despite tending to find their owners on the first trial, they generally switched to a win-stay strategy in subsequent trials, only to return to correctly choosing their owners based on olfaction in the later trials. In Exp 2, the dogs chose randomly throughout. We also found that dogs who relied on visual information in the warm-up trials were less successful in the olfaction-based test. Our results suggest that despite their ability to successfully collect information through olfaction, family dogs often prioritize other strategies to solve basic choice tasks.

## Introduction

The domestic dog has been used for a variety of search related jobs for thousands of years. Both with scents originating from up close, where dogs can effectively detect explosives [1], drugs [2], and even a variety of diseases [3], as well as from scents originating from farther distances, where dogs can be trained to find missing persons [4,5] and track wildlife [6–8], our canine companions continually prove that they have acute olfactory abilities. While it may seem logical that, if dogs reliably use their noses both from up close and from large distances, then they would also use their noses for problem solving at intermediate distances of just a few meters (from which target objects are potentially visible), there have been few studies that have examined whether this is actually the case.

Studies on trained explosive detector dogs suggest that dogs continue using strictly olfactory cues even when the target and other cues are clearly visible to them [9]. It has been claimed that untrained family dogs also behave in the same manner [10], however a number of studies have seen the opposite effect: dogs rely primarily on visual, social, cognitive, and spatial cues rather than olfactory ones [11–16]. These discrepancies in behavior suggest that dogs’ search strategies at short distances may not be as straight forward as one might believe, and that there are a variety of factors that can influence what rule a dog decides to use.

With this in mind, the aim of our study was to assess the strategies used by pet dogs in a spontaneous and natural situation where they needed to determine the location of either their owner or a piece of food from relatively close visually identical positions.

For the owner trials we hid a number of people under thin sheets and tasked the dog with determining which was the owner. We believed this situation to be both analogous to hide-and-seek games often played by owners, as well as, to a certain extent, search tasks that are performed by rescue dogs [17]. In order to assess how the dogs respond to the same setup with direct visual cues (that is, to control for their motivation to approach their visible owners), on a separate sample of ten dogs we first performed a simple experiment with an otherwise similar design (Study 1 Pre-test).

In the food trials, we aimed to mimic the setups of a variety of canine cognitive tests where food is hidden under one of multiple pots (for a review see [18]). In these experiments the procedures controlled for the influence of olfactory cues by two major methods: keeping all the pots the same in respect to potential olfactory cues—either all baited or all empty [14,19–22]–or applying control trials that lacked any visual cues to test for the effect of the possibly more odorous baited pot [12,23].

We performed two studies, each consisting of two experiments: searching for 1) the owner and 2) baited food sequentially. In Study 1, after testing for motivation in a control test with ten dogs, we compared the strategies used by 30 pet dogs in a series of three-way choice tests at 0m, 1m, and 3m to determine the effect of distance on their success. Based on the results of Study 1, in order to exclude a number of confounding factors and alternative explanations, we modified the test design in Study 2 to be a series of two-way choices all at 3m. This was then tested on a new sample of 16 dogs. Both studies sought to answer the question of whether, within a relatively short distance, when the only discriminatory cues available to them were olfactory, dogs would successfully find the correct target. (Due to the fixed order of the experiments, they were not compared directly). As the designs of our studies were setup to assess the different choices dogs make, we aimed to gain a greater understanding not only of dogs’ olfactory abilities, but also of the different strategies they choose to utilize in various natural situations.

Considering that the dogs were shown the correct target even after incorrect choices, as an alternative response we expected them to use some memory-based cognitive strategies, similar to those used when foraging. The most probable strategy we predicted was the “win-stay”, whereby an animal returns to an area where it was previously successful. This strategy is ecologically relevant to many predatory animals, especially in situations where, much like in the setup of our experiments, they are only able to capture a single prey item at one time from a location where prey is repeatedly clustered in consistent patches [24]. For example, many predatory bird species will return to prey on nests where they have found chicks or other small animals in the past, mink will concentrate their fishing efforts to locations where they were previously successful, and foxes will return to kill a second fawn sibling in the same place where they found the first [24,25].

We hypothesized that when the scent would be immediately available to them at 0m, the dogs would make their decisions with their noses and successfully find both the owner and the food. At the farther distances, especially at 3m, they would either be unwilling to make a choice at the starting point and instead wait until they could smell the targets from up close, in which case they would choose randomly, or they would tend to use the win-stay strategy.

### Ethics Statement

In Hungary, the University Institutional Animal Care and Use Committee (UIACUC, Eötvös Loránd University, Hungary) does not require special permissions for non-invasive cognitive studies to be performed on dogs, as Hungarian animal welfare laws do not consider observational procedures to be animal experimentation. All owners and dogs took part in our studies voluntarily and were free to stop participating at any time. Owners signed consent forms where they indicated that they understood the test procedures and agreed to allow their dogs to take part in the study.

## Pre-Test: Control for Motivation

Perhaps the most important condition to be certain of when assessing accuracy in search tasks is the individual’s motivation to find the target. If a subject prefers to approach something novel (for example a stranger rather than an owner), or simply chooses randomly, then not much can be said about locating ability or strategy. For this reason we tested dogs’ general tendency to approach their owners in a task where all the people were visible to the dog (otherwise this was identical to the 3m test trials in Study 1). We did not test the dogs’ motivation for finding the food because the warm-up trials (see later) were able to validate the dogs’ preference for food over no food.

### Methods

The subjects were 10 adult dogs of various breeds (Belgian shepherd dog, border collie, Labrador retriever, medium poodle, Shetland sheepdog, vizsla, and four mixed breeds; 6 male, 4 female; mean age 5.15 ± 3.75) and their owners who were recruited on a volunteer basis from the Family Dog Project Budapest database.

**Fig 1:**
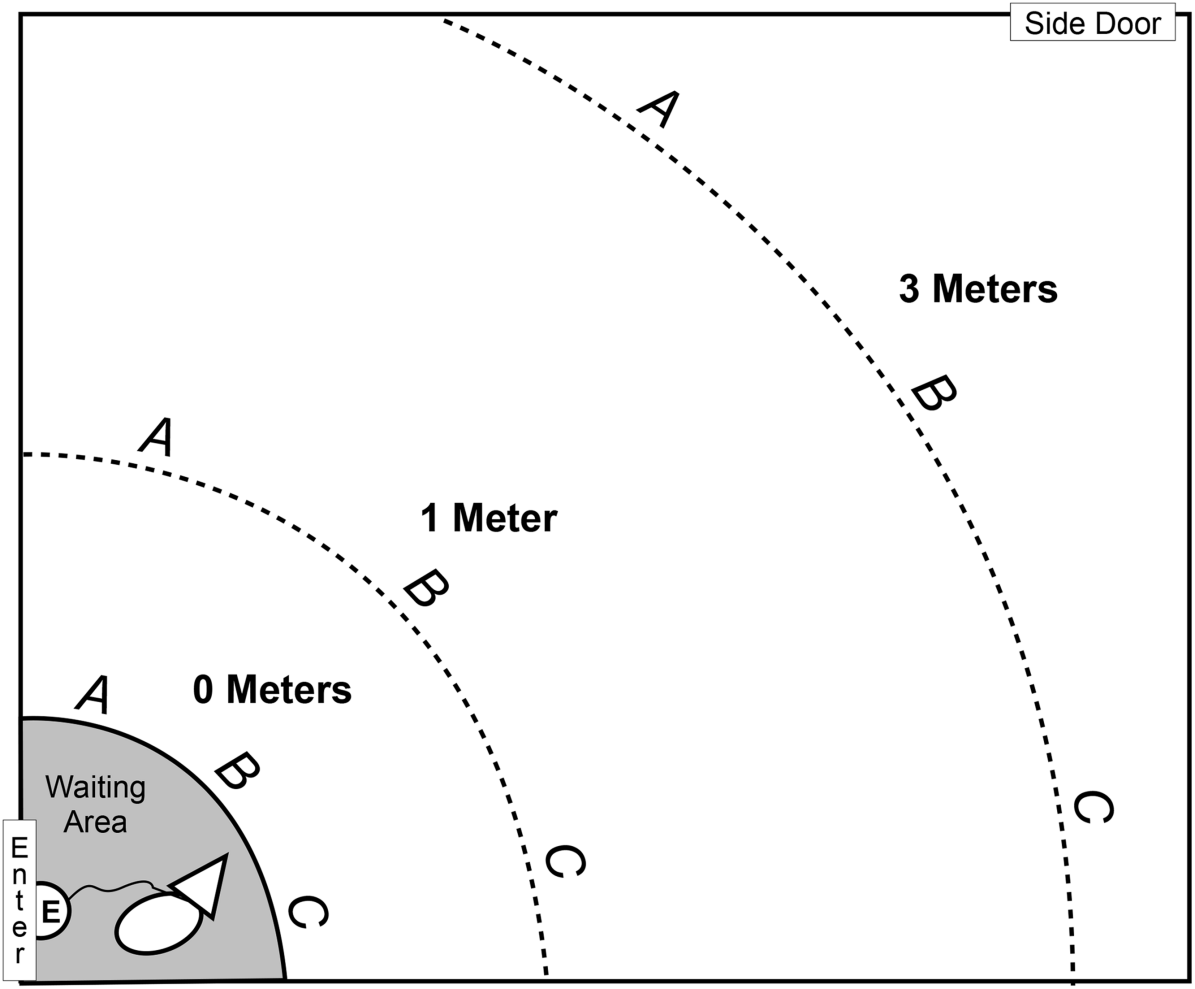
Testing Room. Setup of the testing room with the three conditions (0m, 1m, 3m distances), and three positions (A, B, C). The experimenter (E) and the dog entered the room through the side door during the warm up trials, and through the main door (“Enter”) during the test trials. E and dog stood in the waiting area for 15s at the start of each trial.

The test consisted of three trials, each at a distance of three meters, where the owner and two same-sex helpers sat (fully visible to the dog) in each of the three positions (A, B, C—see Fig 1) once in a randomly determined order. While the experimenter (E) and the dog waited outside, the owner and helpers sat at their predetermined positions. The owner then called the dog to come, at which point E and the dog (on leash) entered the room and waited behind the starting line for 15s. During this period, the three humans were silent and still and looked only at a spot above the door (not at the dog), while E looked only at the stopwatch. After the waiting period, the dog was told, “go ahead,” and was allowed to approach the humans on a long leash. The dogs’ choice was defined as the position the dog initially began walking towards (for the first half of the distance).

The choices were recorded by E by hand (A, B, or C). The average of the successful choices for all the dogs was compared to chance level (1) by a one-sample t-test.

### Results and Discussion

The dogs chose their owners in 96.7% of the trials, which is significantly more than chance level (t_(9)_ = 19, p<0.001). This suggests that at a distance of 3m dogs are not only able to visually determine which of three sitting individuals is their owner, but are also sufficiently motivated to approach him or her over strangers. These results support findings that dogs are able to effectively discriminate visually between their owners and unfamiliar people from a distance, and that they consistently prefer their owners in a variety of situations (e.g. [26]).

## Study 1

### Methods

#### Subjects

The subjects were 30 adult dogs of various breeds (Airedale terrier, boxer, Belgian shepherd dog, Bernese mountain dog, English bulldog, English cocker spaniel, French bulldog, golden retriever (5), Jack Russell terrier (2), Labrador retriever (2), dachshund, pit bull, Siberian husky, Hungarian vizsla (3), mixed breeds (8); 16 male, 14 female; mean age 3.65 ± 1.7 years) and their owners. Seven of the dogs had some training in scent-related work (man-trailing, retrieving/hunting, or tracking). One dog was excluded from Experiment 2 because of a lack of interest in the food.

#### Procedure overview

Study 1 consisted of two experiments, one with the owner (Exp 1) and one with food (Exp 2) run in a fixed order. Testing took place in an empty 4.4m x 3.6m room with two doors, with the air circulation turned off. All sessions were videotaped. The setup was marked on the floor with tape as shown in Fig 1. There were three possible equally spaced positions for the target (A, B, C) in three possible conditions (0m, 1m and 3m). The target was in each location once (position x condition), for a total of nine trials in each experiment. For each trial, the other two positions of the given condition were also filled, either by two helpers who were of the same sex and smoking status as the owner in Exp 1, or by two unbaited pots that were identical to the baited pot in Exp 2. The order of locations was semi-random so that the starting positions and conditions were balanced and neither the same condition nor the same position was repeated on two consecutive trials. The same female experimenter (E) handled all of the dogs. She became familiar with the dogs (so they would not be nervous about being handled by a stranger) by interacting with them while explaining the protocol to the owner prior to the test. During the test, E was blind to the target’s position, which was determined by a preprinted sheet of paper taped to the wall and interpreted by the helpers.

#### Experiment 1- Owner

Before the test, warm-up trials were performed to accustom the dog to the situation. E and the dog waited outside the side door (a different door from where they enter during the test), while the owner hid sitting on the floor under a thin but not transparent sheet (“Night Dreams” brand, beige color, 240x250cm, 100% polyester) at a random position in the room that was not one of the marked positions used in the test. None of the person’s features were visible from under the sheet. When the owner was ready, she called the dog, who was then brought in on leash and allowed to approach the owner. Once the dog spent a few seconds sniffing the sheet, the owner was instructed to show herself and praise the dog. This was repeated, always in a different random location, until the dog freely and immediately approached the sheet (2–4 occasions; twice was usually sufficient).

During the testing trials, the dog and E waited outside the main door (“Enter” in Fig 1) while the owner and two helpers went to their assigned locations. When all three people were hidden under the sheets, the owner called the dog to come. E and the dog (held short on a 3m leash) entered the room and waited in the waiting area for 15s, or, in the case of the 0m condition, until the dog showed clear signs (see later) of having identified the owner. During this time, E looked only at the stopwatch. After the waiting period, the dog was told, “go ahead,” and was allowed to approach the humans while E held the leash from behind the starting line. The dog’s first choice was defined as the position it initially began walking towards within the first half of the tested distance. This criterion was chosen because in the pilot tests we noticed that, if they realized that they had chosen incorrectly, some dogs would switch direction when they were already relatively close to the target, and we were interested in their choices made at the tested distances. After approaching, the dog was allowed to examine all the humans, who remained silent and motionless under their sheets until the dog began displaying signs of owner identification, at which point E said, “okay,” and the sheets were removed. Owner identification was defined as any of the following behaviors: intense tail wagging, jumping, rubbing face into person, trying to remove or crawl under blanket, whining/barking, or remaining within 0.3 meters (or orienting towards in case of condition 0m) of the chosen individual for 5 seconds. Each dog’s personal identification signs were determined during the warm-up trials and considered in the test trials. The owner was allowed to briefly praise the dog before all three humans walked to the back corner of the room. This was done to show the dog that the owner did not remain in the same position. The whole procedure was repeated for each of the nine locations in a semi-random order, where neither the same distance nor the same position was repeated in two consecutive trials.

After Exp 1, there was a 5–15 minute break. The owner filled out a general questionnaire about the dog during this time. Water was provided for the dog.

#### Experiment 2 –Food

The second experiment also began with two warm-up trials. A round plastic flowerpot (18cm wide, 13cm tall, with a 7cm hole cut out of the bottom) was placed over a 15cm wide plastic saucer with a 1cm thick piece of sausage on it (Tesco brand, beef). The dogs were allowed to approach and sniff the pot, which was then lifted, allowing the dog to eat the food. One dog who showed no interest in the food (i.e. did not eat it) was excluded.

The basic setup was the same as in the previous test, but instead of humans, there was one baited pot and two identical unbaited pots placed in the room. The rules for hiding were the same, but different orders were used. When the room was set up, E led the dog to the starting point and the owner stood still behind them. After a 15s waiting period, during which E looked only at the stopwatch, the dog was released on a long leash. The choice criterion was the same as in the previous test. The identification criteria were defined as trying to reach the food (nosing, pawing, pushing at pot) or orienting at one pot for 5 seconds. The dog was only allowed to eat the food if it was in the first approached pot (choice), or, for the 0m condition, if it showed identification signs towards the correct pot within the 15s waiting period. If the dog went to an unbaited pot first, it was still allowed to find the correct pot, but was not allowed to eat the food. This was done so that the dog still had knowledge of where the food was in that trial, but was not rewarded for making an incorrect first choice. Afterwards, the pots were collected in front of the dog, who was then taken into the hallway while the room was reset.

#### Data Collection and Behavioral Coding

Three built-in cameras recorded the room from various angles; a fourth camera was above the waiting area and recorded the position the dog was facing during that time. The initial choices were recorded by hand (A, B, or C). This was then calculated to how many successful choices there were out of the nine trials.

For Exp 1, sniffing time was also recorded as latencies from when the dog first began sniffing a person (nose within ~10cm of the sheet) to when it turned away from said person or began showing signs of identification (Solomon Behavioural Coding Program). Sniffing durations in case of correct vs. false/missed identifications were compared.

#### Data Analysis

The number of correct choices for each condition was compared to chance level (1) through one-sample Wilcoxon signed rank tests. The effect of using the win-stay strategy was analyzed by looking at the number of choices for each condition that was based on the location the target had been in the previous trial. As the very first choice of the nine could never be based on a previous position, this was excluded from the calculation. The number of choices based on win-stay was compared to chance level (8/9 = 0.889) through one sample Wilcoxon tests only in the case of conditions where the dogs’ correct choices were not above chance level.

The effect of previous scent training was analyzed through a Wilcoxon-Mann-Whitney test. To reveal potential within test learning effects, the mean success rate of the first and last four trials (1–4 and 6–9) were compared through a related-samples Wilcoxon signed rank test. The correlation between Exp 1 and Exp 2 was analyzed through Spearman’s rank correlation test.

Inter-observer agreement was assessed on behavior variables using parallel coding for 10 of the 30 dogs. High Cohen Kappa measures were obtained for each variable: sniffing = 0.84, start of identification behaviors (within±2s) = 0.84, and initial choice = 0.95.

### Results and Discussion

#### Experiment 1- Owner

We noticed that in the warm-up trials a number of dogs (n = 11) unexpectedly ran to sniff the opposite door first upon entering the room, rather than their owners who were hiding under the sheets. We found this to be an interesting difference in the dogs’ responses and in light of this, we modified the warm-up procedure in Study 2 to make it possible to assess if there is any association between this behavior and their performances in the test situation (see later).

**Fig 2:**
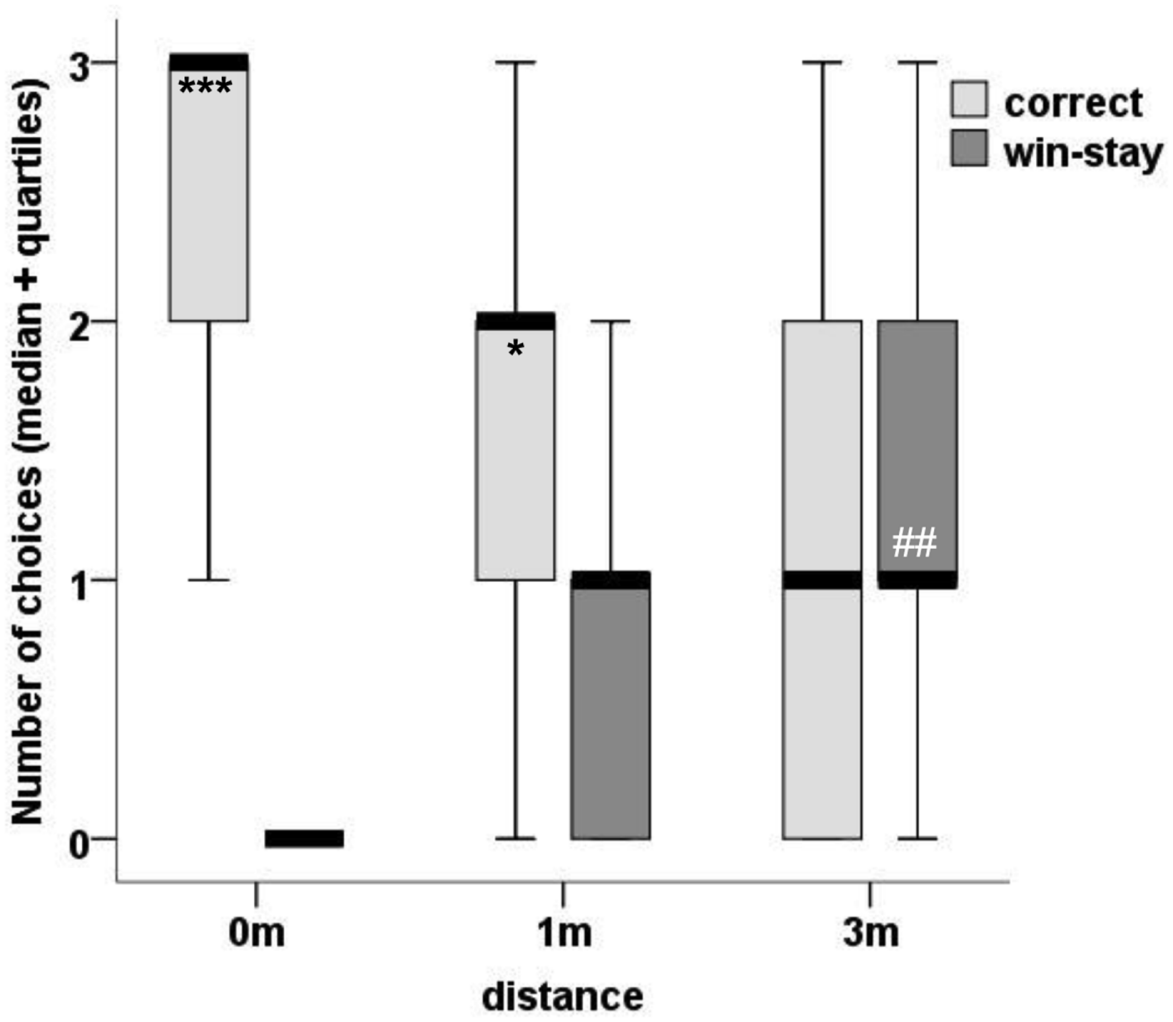
Finding Owner at Three Distances. Median value of choices in each condition that were either successful (correct), or identical to the owner’s position during the previous trial (win-stay). Chance level for success (1 correct out of 3) is 33.3%; *** P<0.001, * P<0.05. Chance level for win-stay is 0.899; ## P<0.01.

In the test trials, the strategies the dogs used to determine which position to approach depended on distance. In the 0m and 1m conditions, the dogs successfully chose their owners (0m: p<0.001; 1m: p = 0.019). In the 3m condition, the dogs did not find their owners more frequently than chance level (p = 0.835). In the case of the unsuccessful 3m conditions, we found that their choices matched with the positions the owners were in the previous trials (p = 0.002) (Fig 2).

As no other cues were available, it appears that at 0m and 1m the dogs were able to pick out their owners based on scent alone, while at 3m the dogs typically made their decisions based on memory. This type of “win-stay” strategy has been observed not only in studies of wild animals, but in socio-cognitive laboratory tests as well (i.e. [27,28]).

There was no evidence of learning, that is, comparing the first four trials to the last four, the performance of the dogs did not improve (Z = -0.747, p = 0.455). There was also no effect of training (Z = -0.275, p = 0.783).

When comparing the average amount of time each of the thirty dogs spent sniffing the different hidden people, the dogs spent less time (mean±SE = 1.9 ± 0.1s) sniffing strangers before moving on than sniffing their owners (mean±SE = 2.7 ± 0.2s) before showing signs of identification (t_(29)_ = 4.47, p<0.001). Furthermore, in the nine instances of false identification, we found that dogs spent 4.4 ± 0.9s sniffing before displaying identification signals. Thus, while the dogs were able to make accurate first choices at 0m and 1m when approaching their owners, they nevertheless spent a significant amount of time sniffing them from up close before displaying identification signals. This may indicate that, despite their acute olfactory abilities, or perhaps on account of them, dogs seem to have a strong preference for smelling from up close, often to the point of actual physical contact. This preference may have an impact on what strategy they decide to choose when searching from various distances.

#### Experiment 2 –Food

The dogs chose the correct pot significantly more than chance in the 0m condition (p<0.001), but not in the 1m (p = 0.491) or 3m conditions (p = 0.491). This unexpectedly low performance can potentially be explained by a number of factors. Compared to some other experiments that used open, right-side-up pots [20,27,28], the 7cm holes on our upside-down pots may have been too small to allow enough scent to come out within such a short time period. In a similar train of thought, we must consider that a 1cm piece of sausage emits significantly less scent than a grown person, thus making it more difficult for the dogs to pick up the scent’s direction. The fix order of the two experiments may also have had an effect on their performance, as the dogs might have simply become fatigued from the previous owner trials and were not performing to the best of their ability, although other similar tests with 18+ trials have not reported any decline in performance in later trials (i.e. [20,28,29]).

**Fig 3:**
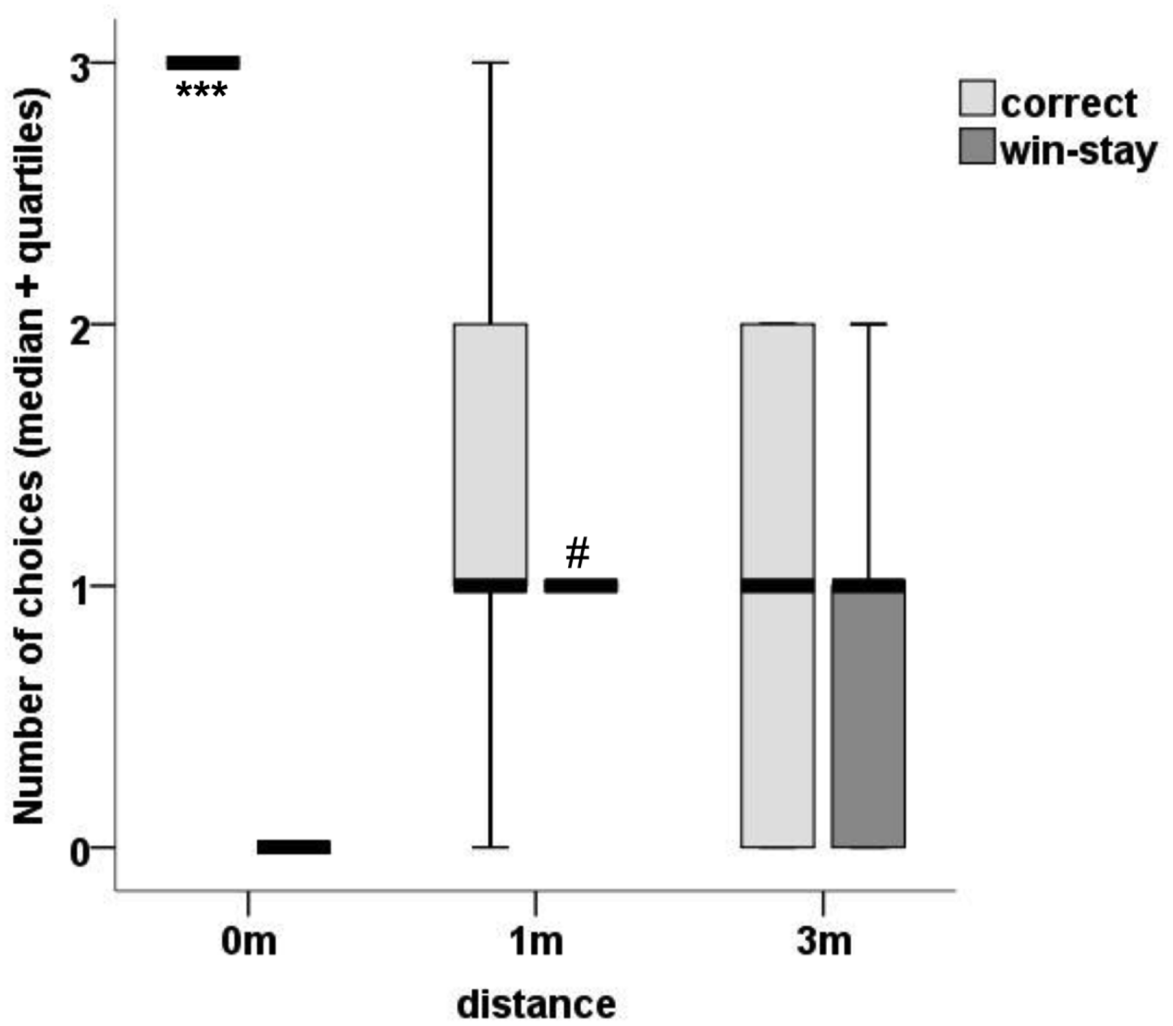
Finding Food at Three Distances. Median value of choices in each condition that were either successful (correct), or identical to the position of the baited food during the previous trial (win-stay). Chance level for success (1 correct out of 3) is 33.3%; *** P<0.001. Chance level for win-stay is 0.899; # P<0.05.

With regards to the win-stay strategy, the dogs based their choices on the food’s previous position slightly above chance for the 1m condition (p = 0.048) but at chance for the 3m condition (p = 0.127) (Fig 3). A plausible explanation for this is that, since the dogs were not allowed to eat the sausage if they did not choose the correct pot on their first try, there was not much “win” upon which to base their “win-stay” strategy. It seems that simply being shown the position of the reward is not as strong of a reinforcer for this effect as being able to find the owner in Exp 1.

There was no evidence of learning between trials 1–4 and 6–9 (Z = -0.755, p = 0.45), nor was there any effect of training (Z = -1.65, p = 0.099).

There was also no correlation between the correct choices in Exp 1 and Exp 2 (r_(27)_ = 0.044, p = 0.821).

## Study 2

In our previous study we found that by a distance of three meters, pet dogs did not accurately choose which of three covered people was their owner or which of three pots was baited with food. However, this result does not necessarily mean that dogs are unable to solve these tasks using their olfactory abilities.

In our second study we aimed to create a simpler version of Study 1 that was more conductive to both potentially olfaction based decisions and to learning. In this version, there were three major differences compared to Study 1: (1) there were only two positions, (2) all trials were performed at 3m, and (3) dogs had 10 trials in each experiment. We assumed that two instead of three targets would make the choice situation more clear-cut, allowing us to exclude the potential overlap of scents in the owner trials. Moreover, we thought the simpler procedure with more trials of the same problem would help the dogs learn how to solve the task. We decided to only test at 3m as this was the distance at which the dogs performed at chance level in both experiments. We also wanted to reveal if it is a characteristic trait of individual dogs to systematically use their noses (successfully) in case of both the owner and food trials.

Furthermore, the food version of this setup was very similar to the one used in the study of Szetei and colleagues [12]. As that study found dogs to be successful in choosing the baited pot in a two-way test at 3m, we hypothesized that with this setup the dogs would, at least in the later trails, perform better than in Study 1.

Lastly, based on our observations in Study 1, we performed the warm-up trials in a more controlled manner where all owners were asked to sit in approximately the same area so as to be able to consistently code their dogs’ responses. We predicted that dogs who would first approach the opposite door, closely passing by their covered owners, might be more likely to use visual cues, concluding that, since they cannot see any people, their owner must have left the room and called them from behind the door. We expected these dogs to use different strategies (not based on scent) during the test situation as well.

### Methods

#### Subjects

The subjects were 16 adult dogs of various breeds (Border collie, dachshund, Doberman, German shepherd, German pointer, golden retriever, Labrador retriever, Nova Scotia duck tolling retriever, sheltie, vizsla, mixed breeds (6); 10 males, 6 females; mean age 4.8 ± 3.6 years) and their owners, recruited on a volunteer basis from the Family Dog Project database. Two dogs were excluded from Exp 1 and another one from Exp 2 due to a lack of interest in the targets.

#### Procedure

This study also consisted of two experiments (searching for the owner and for baited food) run in a fixed order. The procedures for the two experiments were similar to those of Study 1, with only the three differences outlined above, as well as the more standardized warm-up. The positions were renamed “left” and “right” for simplicity, however they were nearly identical to positions “A” and “C” in Study 1. The order of the target’s position was semi-randomly determined so that it was in each position five times but never consecutively in the same position in the first two trials, and not consecutively more than twice in the remaining trials.

#### Data Collection and Analysis

Four cameras recorded the room in the same setup as before. Choices were recorded by hand (left or right), where “choice” was defined using the same criteria as in Study 1. The number of correct choices was compared to chance (5) by one-sample t-tests in Exp 1, one-sample Wilcoxon signed rank tests in Exp 2, and by a binomial test for trials 1 and 2 for both experiments. Due to the potential repetitions of the baited locations (with the exception of the second trial), the use of the win-stay strategy (choices that were based on the location the target had been in the first trial) was not analyzed statistically (but see later). The occurrence of learning was determined by comparing the mean accuracy of Trials 1–5 and Trials 6–10 through a paired t-test in Exp 1 and through a related-samples Wilcoxon signed rank test in Exp 2. The correlation of the number of correct choices between Exp 1 and Exp 2 was analyzed through Spearman’s rank correlation test.

### Results and Discussion

#### Experiment 1 –Owner

During the warm-up trials, eight out of the 14 dogs first examined the door at the other end of the room rather than the owner (see S1 Video). There was a significant difference in the dogs’ success depending on this behavior, where dogs that first examined the opposite door performed significantly worse in the test than dogs who immediately went to their owners (t_(12)_ = 2.4, p = 0.034). This suggests that further studies should investigate what intrinsic tendencies or learnt responses govern the strategies dogs decide to use in such situations.

**Fig 4:**
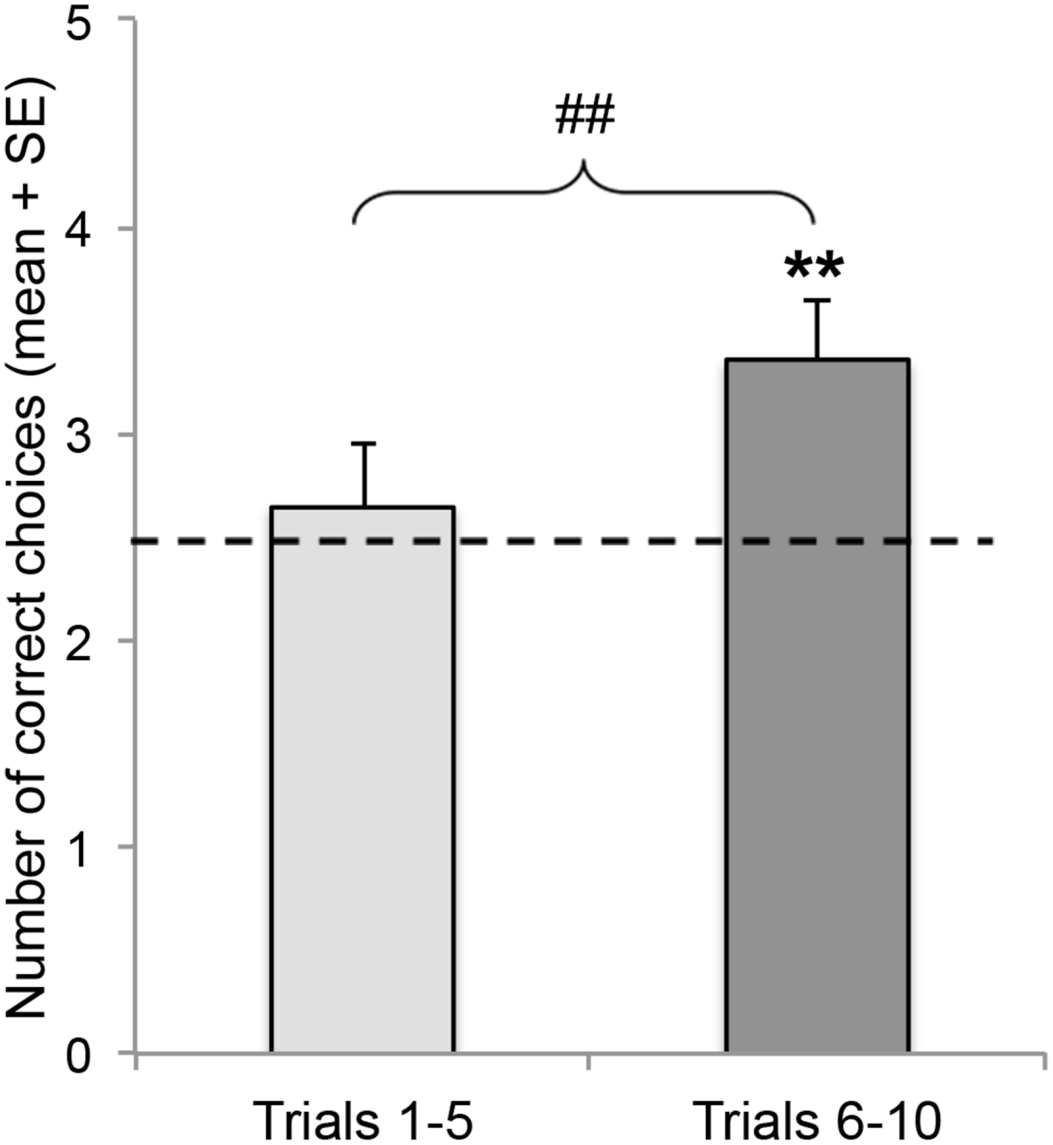
Learning Across Owner Trials. Average number of correct choices in the two halves of the owner trials. **P = 0.01; ## P<0.01. Dashed line represents chance level (2.5 out of 5).

During the test trials, the dogs chose at chance level for the average of the ten trials (t_(13)_ = 1.77, p = 0.1). Although their performance was not better than chance in the first five trials (t_(13)_ = 0.46, p = 0.65), they were able to find their owners better than chance in the final five trials (t_(13)_ = 2.96, p = 0.011). This difference between the two halves of the tests is significant (paired t-test: t_(13)_ = 3.68, p = 0.003) (Fig 4).

**Fig 5:**
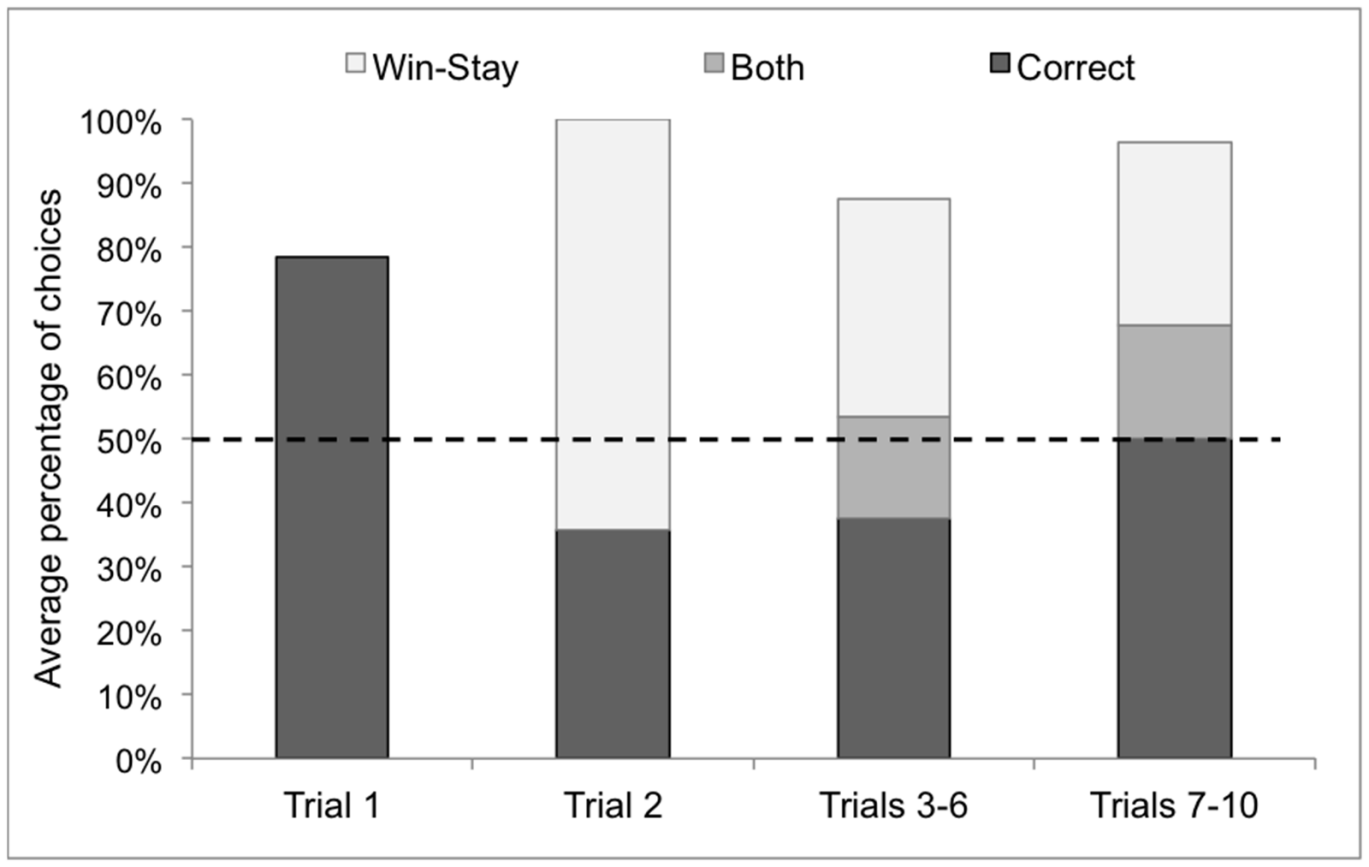
Choices by Trial for Owner Average accuracy and use of win-stay strategy across various owner trials. Dashed line represents chance level for correct choices. Note that in the 2^nd^ trial the target was never positioned to the same place as it was in the 1^st^. From the 3^rd^ trial onward a win-stay choice (choosing the position the owner was located in the previous trial) could be either correct or incorrect.

To reveal the details of this learning effect, we analyzed the data by trial, and found that the dogs did tend to successfully find their owners on the first trial (p = 0.057), however, this level of accuracy decreased to chance level in the second trial (p = 0.424), which was parallel to the emergence of the win-stay strategy, which accounted for 64% of the choices in that trial (Fig 5).

The data suggests that dogs are indeed capable of determining the location of their owners from a distance of 3m by olfaction alone; however, it also shows that with repeated trials many dogs switch to alternative strategies based on information from previous experiences (although it seems that the dogs slowly learned to revert back to olfaction-based solutions once the cognitive strategies regularly proved to be unsuccessful).

#### Experiment 2 –Food

The dogs were not successful in choosing the baited pot at 3m (p = 0.458). They performed at chance level for both the first five trials (p = 0.268), and the last five trials (p = 0.79) and did not show any within test learning (p = 0.351). Furthermore, unlike in Exp 1, the dogs did not have a tendency to choose correctly in the first trial (p = 0.302). In the second trial 67% of the choices matched with the win-stay strategy (see similar results in [28]).

Since in the present studies we aimed to investigate dogs’ inclinations to use their noses in a choice test, our procedure was analogous to the control trials performed in many of the tests investigating dogs’ abilities to rely on human gestural communication (i.e. pointing), with the difference being that in the majority, even those involved an experimenter standing by the pots from whom the dogs could expect some cue. While this could potentially cause the dogs to be less likely to “switch on” their noses, in our tests there was no such interference.

The low level of accuracy found in our test is in contrast to the results of Szetei et al. [12], which, despite having a relatively similar setup where dogs were released from a distance of 3m to choose between a baited and an unbaited pot, found that dogs were able to choose correctly, albeit at a level only slightly above chance. We believe that this discrepancy—besides the minor differences in the procedure—can be due to a simple difference in how a “first choice” was defined in our respective studies. While we coded the first pot that the dog approached (i.e. was walking and orienting towards for the first half of the distance after being released), Szetei et al. defined a choice as the moment the dog “touched the lid of the bowl with the intent to remove it” [12]. This is a key difference, since many dogs were able to smell a bowl from up close without coming into contact with it, and could then move on to examine the other one if they did not detect any meat. Indeed, many of the dogs in our study immediately attempted to approach the other pot as soon as they realized from a few centimeters away that the approached pot was unbaited, which, as mentioned previously, is why we defined the first choice criterion in the manner we did.

Although the dogs’ accuracy did not improve, they also did not tend to use the win-stay strategy in the food trials. As stated previously, this can be due to the fact that simply showing the animal the location of the food (as opposed to allowing it to eat it) when it chooses incorrectly is not rewarding enough to initiate the use of such a strategy. Some support for this can be seen in the second trial where all of the dogs that chose accurately in Trial 1 (n = 5) chose that same side in Trial 2, while only half of the dogs that chose incorrectly in Trial 1 (n = 10) switched. In other words, those dogs that won and ate, stayed with that side (win-stay), while those dogs that lost but were still allowed to see where the food was, continued to choose randomly.

There was no correlation between accuracy in the owner and food results of the same individuals (r_(11)_ = 0.259, p = 0.392).

## General Discussion

Our results suggest that even in situations that can be successfully solved by using olfaction, the strategies that family dogs use vary depending on the distance from the target, as well as the target itself.

Although olfaction is one of the most acute canine senses, it seems that in certain situations, especially when a family dog is presented with the same problem multiple times consecutively, cognitive strategies can take precedence over olfaction, even when smelling has proven a successful solution in the past. The switch to this strategy is likely highly dependent on the positive experiences that occur at a given position, as demonstrated by the fact that the win-stay strategy did not manifest as greatly in the food trials where the animals were not allowed to eat the sausages if they chose incorrectly, as opposed to the owner trials where they could always find (and greet) the owner, (although without further study we cannot exclude that this difference is due to some other difference between how dogs view each target—for example that they do not have permission to eat food at certain locations).

Our results match the majority of those found in the control tests for studies looking at the effect that social cues have on a dog’s ability to locate hidden food. In those tests, much like in our studies, the dogs did not choose the baited pot over the unbaited pot(s) in the absence of social or other visual cues [21,23,29,30]. It has been suggested that these control trials are not sufficient in excluding the possibility that dogs identify the baited containers through olfaction alone [10], however, the data beyond this claim are unpublished. We believe that our results provide sufficient validation to the non-social control tests of studies with similar set ups.

While the primary aim of our study was not to assess scenting ability, but rather to analyze the strategies used, the insights gained from our results will certainly be applicable to many future studies that do aim to measure olfactory capacities. For example, although we tried to exclude any salient visual cues, dogs nevertheless first attempted to solve the problems based on the little visual information they had, rather than on the available olfactory cues. In the warm-up trials of Study 2/Exp 1, the majority of the dogs ran expectantly to examine the door on the opposite side of the room after entering. A plausible explanation could be that, based on the visual information they gained upon entering the room, the dogs determined that their owners must have left the room and called them from behind the door, rather than from the sheet under which the owner was hiding, and past which, incidentally, the dogs had to run to get to the door. Thus, if one wants to assess the actual abilities of canine olfaction in a search situation, it is important to remember that many untrained family dogs seem to first focus on solutions based on other sensory cues before “turning on” their noses, even if the target is right next to them.

With this in mind, one has to wonder how much of an impact having visual choices had on what strategy the dogs decided to choose in our studies. It is entirely possible that, due to a preference for sniffing from up close rather than from many meters way, the dogs were simply waiting to be released so that they could sniff each option one by one, as they already knew that the target must be in one of the visible positions. It would be interesting to see in future studies whether dogs use different strategies and are more accurate in a setup where they are unable to see the targets and thus must determine their initial direction by actually searching rather than just choosing. Therefore, our results may not be reflective of dogs’ olfactory capabilities and it would be erroneous to conclude, based on our data, that dogs are incapable of solving these tasks through olfaction.

In summary, it seems that family dogs tend to use rule-based strategies parallel to and sometimes prioritized over olfactory ones in repeated choice designs. Even when an olfactory strategy has proven successful, like for the majority of dogs in the first owner trial of Study 2, they still choose to base the decision of the next trial on the previous experience rather than on using their noses again (although, many dogs do seem to return to olfaction-based solutions later on if cognitive strategies prove unsuccessful over time). This brings into question the commonly held notion that dogs rely first and foremost on their noses for solving these sorts of search problems. Of particular note is our result that even at a distance of just one meter, dogs did not choose to first approach the pot baited with sausage over the unbaited ones. This unexpected result is a clear indication that more research is needed into how dogs use their various senses to perceive and behave in our world, especially with regards to their olfactory abilities and search strategies.

## Supporting Information

S1 Dataset Data Used for Statistical Analysis. (XLSX)

S1 Video Example of Visual Strategies Used by Dogs in Warm-Up. Many dogs ran past their hidden owners to first examine the door during the warm-up trials.
(MOV)

## References

[1] Furton K, Myers L. The scientific foundation and efficacy of the use of canines as chemical detectors for explosives. Talanta. 2001;54:487–500. 18968273 10.1016/s0039-9140(00)00546-4

[2] Jezierski T, Adamkiewicz E. Efficacy of drug detection by fully-trained police dogs varies by breed, training level, type of drug and search environment. Forensic Sci Int. 2014;237:112–8. 10.1016/j.forsciint.2014.01.013 24631776

[3] Bijland L, Bomers M, Smulders Y. Smelling the diagnosis A review on the use of scent in diagnosing disease. Neth J Med. 2013;71(6):300–7. 23956311

[4] Fenton V. The use of dogs in search, rescue and recovery. J Wilderness Med. Elsevier; 1992 8;3(3):292–300.

[5] Wells DL, Hepper PG. Directional tracking in the domestic dog, Canis familiaris. Appl Anim Behav Sci. 2003 12;84(4):297–305.

[6] Cablk ME, Sagebiel JC, Heaton JS, Valentin C. Olfaction-based detection distance: A quantitative analysis of how far away dogs recognize tortoise odor and follow it to source. Sensors. 2008;8(4):2208–22. 27879818 10.3390/s8042208 PMC3673414

[7] Reed SE, Bidlack AL, Hurt A, Getz WM. Detection distance and environmental factors in conservation detection dog surveys. J Wildl Manage. 2011 1 31;75(1):243–51.

[8] Rolland RM, Hamilton PK, Kraus SD, Davenport B, Gillett RM, Wasser SK. Faecal sampling using detection dogs to study reproduction and health in North Atlantic right whales (Eubalaena glacialis). J Cetacean Res Manag. 2006;8(2):121–5.

[9] Gazit I, Terkel J. Domination of olfaction over vision in explosives detection by dogs. Appl Anim Behav Sci. 2003 6;82(1):65–73.

[10] Udell M a. R, Wynne CDL. Ontogeny and phylogeny: both are essential to human-sensitive behaviour in the genus Canis. Anim Behav. Elsevier Ltd; 2010 2;79(2):e9–14.

[11] Dumas C. Figurative and spatial information and search behavior in dogs (Canis familiaris). Behav Processes. 1998 2;42(2–3):101–6. 24897457 10.1016/s0376-6357(97)00071-5

[12] Szetei V, Miklósi Á, Topál J, Csányi V. When dogs seem to lose their nose: an investigation on the use of visual and olfactory cues in communicative context between dog and owner. Appl Anim Behav Sci. 2003 9;83(2):141–52.

[13] Horowitz A, Hecht J, Dedrick A. Smelling more or less: Investigating the olfactory experience of the domestic dog. Learn Motiv. 2013;44(4):207–17.

[14] Macpherson K, Roberts W a. Spatial memory in dogs (Canis familiaris) on a radial maze. J Comp Psychol. 2010 2;124(1):47–56. 10.1037/a0018084 20175596

[15] Gagnon S, Dore F. Search behavior in various breeds of adult dogs (Canis familiaris): Object permanence and olfactory cues. J Comp Psychol. 1992;106(1):56–68. 10.1037/0735-7036.106.1.58 1365009

[16] Salvin HE, McGreevy PD, Sachdev PS, Valenzuela MJ. The canine sand maze: an appetitive spatial memory paradigm sensitive to age-related change in dogs. J Exp Anal Behav. 2011 1;95(1):109–18. 10.1901/jeab.2011.95-109 21541168 PMC3014775

[17] Shivik JA. Odor-adsorptive clothing, environmental factors, and search-dog ability. Wildl Soc Bull. 2002;30(3):721–7.

[18] Miklósi Á, Soproni K. A comparative analysis of animals’ understanding of the human pointing gesture. Anim Cogn. 2006;9(2):81–93. 16235075 10.1007/s10071-005-0008-1

[19] Pongrácz P, Gácsi M, Hegedüs D, Péter A, Miklósi Á. Test sensitivity is important for detecting variability in pointing comprehension in canines. Anim Cogn. 2013;16(5):721–35. 10.1007/s10071-013-0607-1 23392852

[20] Miklósi Á, Polgárdi R, Topál J, Csányi V. Use of experimenter-given cues in dogs. Anim Cogn. 1998;1(2):113–21. 10.1007/s100710050016 24399275

[21] Plourde V, Fiset S. Pointing gestures modulate domestic dogs’ search behavior for hidden objects in a spatial rotation problem. Learn Motiv. Elsevier Ltd; 2013 11;44(4):282–93.

[22] Udell M a. R, Dorey NR, Wynne CDL. Wolves outperform dogs in following human social cues. Anim Behav. 2008 12;76(6):1767–73.

[23] Bräuer J, Kaminski J, Riedel J, Call J, Tomasello M. Making inferences about the location of hidden food: social dog, causal ape. J Comp Psychol. 2006 2;120(1):38–47. 16551163 10.1037/0735-7036.120.1.38

[24] Sonerud GA. Brood movements in grouse and waders as defence against win-stay search in their predators. Oikos. 1985;44(2):287–300.

[25] Panzacchi M, Linnell J, Odden M, Odden J, Andersen R. Habitat and roe deer fawn vulnerability to red fox predation. J Anim Ecol. 2009;78(6):1124–33. 10.1111/j.1365-2656.2009.01584.x 19563469

[26] Kerepesi A, Dóka A, Miklósi Á. Dogs and their human companions: The effect of familiarity on dog-human interactions. Behav Processes. Elsevier B.V.; 2014 2 16; 10.1016/j.beproc.2014.02.005 24548652

[27] Ashton R, De Lillo C. The role of association and of human informative gestures in the control of spatial search in domestic dogs (Canis familiaris). J Vet Behav Clin Appl Res. 2009;4(2):50–1.

[28] Gácsi M, Kara E, Belényi B, Topál J, Miklósi Á. The effect of development and individual differences in pointing comprehension of dogs. Anim Cogn. 2009 5;12(3):471–9. 10.1007/s10071-008-0208-6 19130102

[29] Hare B, Tomasello M. Domestic dogs (Canis familiaris) use human and conspecific social cues to locate hidden food. J Comp Psychol. 1999;113(2):173–7.

[30] Lakatos G, Gácsi M, Topál J, Miklósi Á. Comprehension and utilisation of pointing gestures and gazing in dog-human communication in relatively complex situations. Anim Cogn. 2012 3;15(2):201–13. 10.1007/s10071-011-0446-x 21927851

